# Wheat germ cell-free expression system as a pathway to improve protein yield and solubility for the SSGCID pipeline

**DOI:** 10.1107/S1744309111032143

**Published:** 2011-08-31

**Authors:** Katherine Guild, Yang Zhang, Robin Stacy, Elizabeth Mundt, Sarah Benbow, Amanda Green, Peter J. Myler

**Affiliations:** a Seattle Biomedical Research Institute, USA; b Department of Global Health, University of Washington, USA

**Keywords:** cell-free expression, protein expression, protein solubility, Seattle Structural Genomics Center for Infectious Disease, NVoy, *E. coli* expression

## Abstract

Recombinant expression of proteins of interest in *Escherichia coli* is an important tool in the determination of protein structure. However, lack of expression and insolubility remain significant challenges to the expression and crystallization of these proteins. The SSGCID program uses a wheat germ cell-free expression system as a rescue pathway for proteins that are either not expressed or insoluble when produced in *E. coli*. Testing indicates that the system is a valuable tool for these protein targets. Further increases in solubility were obtained by the addition of the NVoy polymer reagent to the reaction mixture. These data indicate that this eukaryotic cell-free expression system has a high success rate and that the addition of specific reagents can increase the yield of soluble protein.

## Abstract 2

A set of 44 protein targets was used to test expression in the wheat germ cell-free system, the vast majority of which were expressed and soluble in this system; further increases in solubility were achieved by addition of the NVoy polymer.

## 1. Introduction

### 1.1. Protein expression

An impediment to the successful production of large quantities of natively folded proteins in *Escherichia coli* is the tendency of many proteins to become insoluble when overexpressed. In order to successfully produce quantities of these proteins sufficient for crystallization, additional methods are necessary; *in vitro* systems using other organisms as well as cell-free systems utilizing extracts from prokaryotic or eukaryotic organisms have been developed (Gräslund *et al.*, 2008 ▶; Endo & Sawasaki, 2006 ▶). Eukaryotic systems utilize a protein-folding apparatus that has evolved to direct the folding of more complex proteins, while cell-free systems are not dependent on the survival of a cell (Klammt *et al.*, 2006 ▶).

Prokaryotic cell-free systems exist, although these systems have demonstrated limited improvement over prokaryotic *in vivo* methods; the misfolding of proteins remains a significant problem (Hillebrecht & Chong, 2008 ▶). The wheat germ cell-free expression system combines the advantages of cell-free and eukaryotic systems and is well suited for expression of difficult-to-express proteins such as disulfide-bond-containing or integral membrane proteins (Endo & Sawasaki, 2006 ▶; Kawasaki *et al.*, 2003 ▶; Spirin, 2004 ▶; Vinarov, Loushin Newman & Markley, 2006 ▶; Vinarov, Loushin Newman, Tyler *et al.*, 2006 ▶; Klammt *et al.*, 2006 ▶; Tyler *et al.*, 2005 ▶). This system has been used as a rescue pathway for human proteins that are not soluble in both *in vivo* and *in vitro* *E. coli* systems (Langlais *et al.*, 2007 ▶).

A recent analysis of *in vivo* and *in vitro* expression of *Arabidopsis thaliana* proteins found that 95–97% of a set of protein targets were soluble when expressed *via* a wheat germ cell-free system in com­parison to 40% when expressed using the *E. coli* cell-based system (Langlais *et al.*, 2007 ▶). These two systems were also tested on a *Plasmodium falciparum* protein set and while detectable protein was obtained for 30% of the proteins in *E. coli*, protein was obtained for 75% when expressed in the eukaryotic cell-free system (Tyler *et al.*, 2005 ▶). Thus, eukaryotic *in vitro* systems, specifically the wheat germ cell-free system, hold significant promise.

### 1.2. Solubility

There is extensive literature on the variables leading to insoluble recombinant expression of proteins. Protein aggregation remains a significant problem in *E. coli* expression systems. Tags used to purify proteins often affect the solubility, and the addition of various tags can lead to the soluble expression of a previously insoluble protein (Gordon *et al.*, 2008 ▶; Ohana *et al.*, 2009 ▶; Sun *et al.*, 2011 ▶). Modification of the sequence, such as the addition of highly acidic sequences, can also solubilize a previously insoluble protein (Zhang *et al.*, 2004 ▶) and different tags may affect solubility (Widakowich *et al.*, 2011 ▶). Proteins with similar features in their native sequences may have a greater tendency towards solubility when recombinantly expressed *in vivo*. The frequency of individual amino acids as well as the frequency of different types of amino acids within a protein has been shown to affect *in vivo* solubility in *E. coli*. It is likely that secondary structure also plays a role in protein solubility and the tendency to form amyloid bodies *in vivo* (Idicula-Thomas & Balaji, 2005 ▶, 2007 ▶). If the protein produced in *E. coli* is primarily insoluble, denaturing and refolding can be attempted. Common denaturing reagents include guanidinium and urea. The refolding process can be aided by the addition of stabilizing agents such as l-arginine (Kudou *et al.*, 2011 ▶). Cell-free systems have an additional advantage in the production of soluble protein, as agents that aid in protein folding can be directly added to the translation reaction. Eukaryotic expression systems, including the wheat germ cell-free system, have been demonstrated to raise the solubility of a protein (Dadashipour *et al.*, 2011 ▶; Klammt *et al.*, 2006 ▶; Langlais *et al.*, 2007 ▶).

## 2. Materials and methods

### 2.1. Protein selection

Proteins in this analysis are a subset of protein targets entered into the Seattle Structural Genomics Center for Infectious Disease (SSGCID) pipeline. The target organisms were NIAID category A–C pathogens. Proteins within target organisms were selected bioinformatically for homology to current drug targets or nominated for structure determination by the scientific community. Proteins were eliminated if they contained more than eight cysteines or a predicted transmembrane domain in the absence of a signal peptide. A total of 44 proteins were used in this analysis, a summary of which can be found in Table 1 ▶. Most of the proteins in this set ‘failed’ expression or solubility testing in *E. coli*; targets were classified as ‘failed’ if low levels of soluble protein were obtained on expression in the standard *E. coli* conditions for SSGCID (Myler *et al.*, 2009 ▶).

### 2.2. Cloning

DNA of the target proteins was cloned into the pAVA0421 vector *via* ligation-independent cloning (LIC) and grown on LB–carbenicillin plates. The pAVA0421 vector contains an N-terminal hexahistidine affinity tag (MAHHHHHH) for imobilized metal-ion affinity chromatography (IMAC). Plasmids were purified using a GenElute HP Plasmid Mini-Prep Kit (Sigma–Aldrich, Dallas, Texas, USA) and transformed into *E. coli* BL21 (DE3) Rosetta cells (EMD Chemicals, San Diego, California, USA) for expression screening. Small-scale protein expression was carried out and evaluated by Western blotting. All constructs were sequenced in the forward direction to confirm that the correct protein target had been cloned.

DNA templates were obtained from the SSGCID pipeline (Myler *et al.*, 2009 ▶). Following *E. coli in vivo* expression trials, PCR products of the target gene including the six-His tag were amplified from the pAVA0421 vector. The PCR products were then cloned into the cell-free expression vector pEU-E01-LIC1 (pEU-LIC), which had previously been modified to accommodate ligation-independent cloning. Targets were PCR-amplified from the prokaryotic expression vector with RedTaq (Sigma, St Louis, Missouri, USA) using the primers F, CTCACCACCACCACCACCATATG, and R, ATCCTATCTTACTCACTTAGCAGCCGGATCCTCGAG, inserted into pEU-LIC using ligation-independent cloning and transformed into Top10 cells (Invitrogen, Carlsbad, California, USA), which were then grown on LB–carbenicillin plates. Individual colonies were screened for insertion *via* colony PCR. DNA from the positive clones was maxi-prepped (Sigma, St Louis, Missouri, USA) and the full insert was sequenced in both the forward and reverse directions to confirm that the correct sequence had been cloned and that the insert was free of mutations.

### 2.3. Expression and solubility testing

Transcription reactions for small-scale screening were performed in PCR strip tubes. In each of the reaction tubes, 2 µg plasmid DNA was mixed with transcription buffer (80 m*M* HEPES–KOH pH 7.8 containing 20 m*M* MgCl_2_, 2 m*M* spermidine hydrochloride, 10 m*M* dithiothreitol), 3 m*M* NTP mix, 2.4 U µl^−1^ SP6 RNA polymerase and 1.2 U µl^−1^ RNase inhibitor; RNase-free water was used to bring the final volume to 20 µl. Transcription reactions were then incubated for 4–6 h at 310 K. A Microcon YM-30 filter (Millipore, Billerica, Massachusetts, USA) was used for small-scale mRNA clean-up.

Small-scale translation reactions were performed in 96-well plates and synthesized RNA was added to the translation mixture; large-scale reactions were performed using either the Protemist DT II robot (Cell Free Sciences, Yokohama, Japan), which also performs sequential transcription, translation and purification steps, or the Protemist XE robot (Cell Free Sciences, Yokohama, Japan), a robot that performs continuous translation for high yields of protein production. Small-scale and large-scale translations were performed using WEPRO1240H (Cell Free Sciences, Yokohama, Japan) cell extract according to the manufacturer’s instructions. For large-scale purification, the translation mixture was clarified by centrifugation at 6000 rev min^−1^ for 30 min at 277 K. The supernatant was purified by IMAC using a HisTrap FF 5 ml column (GE Biosciences, Piscataway, New Jersey, USA) equilibrated with binding buffer consisting of 25 m*M* HEPES pH 7.0, 300 m*M* NaCl, 5% glycerol, 30 m*M* imidazole, 1 m*M* tris(2-carboxyethyl)phosphine (TCEP) and eluted with 500 m*M* imidazole in the same buffer. Concentrated pure protein was flash-frozen in liquid nitrogen and stored at 193 K. Additional translations were performed using WEPRO7240H extract (Cell Free Sciences, Yokohama, Japan) according to the manufacturer’s instructions. For selected protein targets, NVoy (also known as NV10), a commercially available linear carbohydrate-based polymer of 5 kDa molecular weight (Expedeon, San Diego, California, USA), was used to improve solubility yields. NVoy was added directly to the translation-reaction mixture in the Protemist XE system at a concentration of 1 mg ml^−1^. Solubility was assessed in the presence and the absence of the NVoy polymer.

From the translation reaction, two 10 µl aliquots were taken. The total protein from the first aliquot was mixed with 10 µl sample buffer. The other aliquot was spun in a microcentrifuge at top speed for 60 s; the supernatant (soluble) was separated from the pellet and mixed with 10 µl sample buffer, while the pellet (insoluble) was resuspended in 20 µl sample buffer. All samples were boiled at 368 K for 10 min and 10 µl of each sample was loaded onto a gradient SDS–PAGE gel (Pierce Bioscience, Rockford, Illinois, USA) for analysis. Gels were stained with SimplyBlue SafeStain (Invitrogen, Carlsbad, California, USA) and expression levels were based on visual inspection of the SDS–PAGE gels and scaling the intensity of the expected protein bands. The identities of the hexahistidine-tagged proteins were confirmed by Western blotting using an anti-His antibody (Qiagen, Valencia, California, USA). In addition to confirming that the protein was of the correct size, the protein molecular-weight standard (Bio-Rad, Hercules, California, USA) was used as a reference for the quantity of His-tagged protein. Briefly, the approximate quantity of protein in each marker band was estimated and the band intensities of the His-tagged proteins were then compared with the marker proteins. A ‘high’ expression score (+++) corresponded to greater than 0.75 µg µl^−1^ target protein in the reaction mixture. A ‘medium’ expression rating (++) corresponded to more than 0.30 µg µl^−1^ but less than 0.75 µg µl^−1^ and a ‘low’ expression rating (+) corresponded to a visible band of less than 0.30 µg µl^−1^. A ‘no detectable protein’ rating (−) corresponded to no detectable expression on either SDS–PAGE or Western blot gels.

Ratings of the solubility were also determined by visual inspection of the protein bands on SafeStained SDS–PAGE gels using a system similar to that employed for the scoring of expression. The ratio of the band intensities resulting from the pairs of soluble and insoluble proteins was used to rate solubility. A high solubility score (+++) was assigned when 75–100% of the total protein was in the soluble fraction. A medium solubility rating (++) was assigned when the soluble and the insoluble fractions were approximately equal in band intensity. A low solubility rating (+) was assigned when less than 25% of the total protein intensity was in the soluble fraction. Samples with an absence of detectable soluble protein were assigned an insoluble rating (−).

## 3. Results

### 3.1. Small-scale expression

A total of 44 protein targets were analyzed. Two were from eukaryotic organisms (*Trypanosoma brucei* and *Leishmania infantum*); the remainder originated in prokaryotes (the genera *Anaplasma*, *Bartonella*, *Borrelia*, *Brucella*, *Burkholderia*, *Ehrlichia* and *Mycobacterium*). In this set, 20% of the protein targets were soluble when expressed in *E. coli*, 52% were insoluble and the remaining 22% were not expressed (Table 1 ▶). The first stage of the wheat germ cell-free expression pipeline was designed for small-scale screening. During these tests, detectable protein was achieved for 87% of targets based on Western blot analysis (Tables 1 ▶ and 2 ▶). 83% of the targets expressing insoluble protein in *E. coli* expressed soluble protein in the cell-free system (Table 1 ▶). These data demonstrate that the wheat germ cell-free expression system is effective at production of soluble protein.

### 3.2. Large-scale expression

A collection of 29 proteins were selected from the first set of robust small-scale-screened targets for further scaling up in the Protemist DT II using WEPRO1240H extract. (Transcription and translation reactions as well as affinity purification are performed sequentially in the robot.) From this set, 28 targets produced detectable protein, with medium to high quantities of protein for 17 of these targets (Table 2 ▶). Additionally, the small-scale expression testing successfully predicted the expression level and degree of solubility of proteins produced on the large scale in the DT II robot (data not shown). Of the 29 targets, five with varying levels of solubility were selected for testing in the Protemist XE robot with WEPRO7240H cell-free extract, which has been optimized for high-level expression of His-tagged proteins using the Protemist XE (Table 3 ▶). Of the five proteins tested in this system, large quantities of protein were obtained for four (Table 3 ▶, data not shown), although only small quantities of the protein were soluble. Small quantities of the fifth protein were produced, but these quantities were sufficient for crystallization using the microcapillary method (Gerdts *et al.*, 2008 ▶; Yadav *et al.*, 2005 ▶). As a result, additional methods were necessary to produce significant quantities of soluble protein.

### 3.3. Solubility testing

One of the more prominent challenges in the WEPRO7240H expression system is the tendency of protein products to form precipitates: some runs of the Protemist XE resulted in a mixture so turbid that the visible-light sensor was unable to function correctly. Likewise, large-scale expression using WEPRO1240H with the Protemist XE resulted in increased protein yields accompanied by a decrease in solubility (data not shown). One of the advantages of the *in vitro* system is the ability to add reagents as necessary, such as those that help to increase solubility, directly to the translation reaction. One of the factors contributing to protein aggregation is the interaction of exposed hydrophobic patches owing to incorrect protein folding. We therefore chose to investigate the use of NVoy polymer in the cell-free system. Nvoy consists of a carbohydrate backbone with hydrophobic side chains which mask any hydrophobic patches on the protein, thereby limiting nonspecific interactions which can cause aggregation. The effects of the NVoy polymer on solubility and its impact on expression levels were examined in a series of experiments carried out in the Protemist DT II on a subset of five protein targets (Table 2 ▶). This subset consisted of targets for which less than 75% of the proteins were soluble when expressed in the DT II or XE; when expressed in *E. coli*, the protein was predominantly insoluble. WEPRO1240H in the presence and absence of NVoy was tested for five proteins and WEPRO7240H in the presence and absence of NVoy was tested for five proteins, four of which were also used in the WEPRO1240H testing. The NVoy polymer did not decrease the solubility of any of the proteins tested. For two proteins, the expression levels were lower in the presence of NVoy, but this decrease in expression level was accompanied by an increase in solubility (data not shown, Table 3 ▶). One protein, which was com­pletely insoluble when expressed with WEPRO7240H, was over 75% soluble when expressed in the same system in the presence of NVoy. Overall, in seven of the 11 paired NVoy(−)/NVoy(+) com­parisons the NVoy reagent increased the percentage soluble protein yield and in half the experiments the reagent more than doubled the yield of soluble protein; for one protein, we were able to obtain more than 1 mg ml^−1^ soluble protein (Table 3 ▶). These tests demonstrate that NVoy does not substantially reduce total protein yields and in most tests increased the quantity and the percentage of soluble protein produced.

## 4. Discussion

This analysis of expression of proteins from the SSGCID pipeline in a eukaryotic *in vitro* system validates the use of the wheat germ cell-free system for expression of proteins that are either not expressed or are primarily insoluble when expressed in *E. coli*. The quantities of soluble protein were sufficient for microcapillary crystallization as developed by Emerald BioSystems (Gerdts *et al.*, 2008 ▶; Yadav *et al.*, 2005 ▶), although no crystal structures of any of the protein targets in this set have yet been obtained. Combining the wheat germ cell-free expression system with a crystallization method such as microcapillary crystallization may yield structural data for proteins that have been difficult to express.

A lingering concern is the tendency for insolubility to increase as the quantity of protein produced increases. As a result, for most targets similar quantities of soluble protein were obtained from the use of WEPRO1240H (optimized for the DT II) and WEPRO7240H (optimized for high-level expression in Protemist XE) in the absence of additional solubilizing agents. This may reflect the tendency of the protein to form insoluble aggregates at higher concentrations. The addition of NVoy substantially increased the quantities of soluble protein produced, although even in the presence of NVoy the quantities of soluble protein were insufficient for standard crystallization studies. This demonstrates that NVoy can be added to the translation reaction to increase solubility, potentially eliminating the need to denature and refold the protein for solubility. The addition of other solubilizing reagents may lead to further increases in solubility.

These data demonstrate the utility of the wheat germ cell-free system for expression of proteins that are insoluble when expressed in *E. coli*. The addition of NVoy substantially increased the yield of soluble protein; this reagent is likely to increase the production of soluble proteins that are insoluble in *E. coli*.

## Figures and Tables

**Table 1:** Protein set used in this analysis Solubility in *E. coli* reflects testing; ND, no data. Expression and solubililty ratings reflect small-scale expression using WEPRO1240H. Expression key: −, less than 15 µg µl^−1^; +, less than 0.30 µg µl^−1^; ++, less than 0.75 µg µl^−1^ but greater than 0.30 µg µl^−1^; +++ greater than 0.75 µg µl^−1^ Solubility key: −, no soluble protein; +, less than 25% total protein soluble; ++, 25–75% total protein soluble; +++, greater than 75% total protein soluble.

Species	Accession ID	Sequence cloned	Annotation	Solubility in *E. coli in vivo*	Cell-free expression	Cell-free solubility
*Anaplasma marginale*	ACR67103.1	Full length	Major surface protein 2 variant 9H1	ND	+	−
*Anaplasma marginale*	ACR67104.1	Full length	Major surface protein 2	ND	−	−
*Anaplasma marginale*	ACR67105.1	Full length	Major surface protein 2 variant E6F7/1/2	ND	+	−
*Bartonella henselae*	YP_033889.1	Full length	UDP-3-*O*-(3-hydroxymyristoyl) *N*-acetylglucosamine deacetylase	Insoluble	+++	ND
*Bartonella henselae*	YP_032889.1	Full length	ABC transporter/ATP-binding protein	Insoluble	++	++
*Bartonella henselae*	YP_034187.1	Full length	Holliday junction DNA helicase B	Insoluble	++	+
*Bartonella henselae*	YP_033595.1	Full length	DNA topoisomerase IV subunit B	Insoluble	+	−
*Bartonella henselae*	YP_033416.1	Full length	Hypothetical protein	Insoluble	++	−
*Borrelia burgdorferi*	NP_212600.1	Full length	Holliday junction DNA helicase B	No expression	++	++
*Brucella melitensis*	YP_419002	Full length	Catalase	Insoluble	++	+
*Brucella melitensis*	YP_419049	Full length	ATP/GTP-binding site motif A (P-loop):ABC transporter:AAA ATPase:TOBE domain	Insoluble	ND	ND
*Brucella melitensis*	YP_414617	Full length	ATP/GTP-binding site motif A (P-loop):ABC transporter:AAA ATPase:TOBE domain	No expression	++	++
*Brucella melitensis*	YP_414349	Full length	Acetyl-CoA carboxylase, biotin carboxylase:carbamoyl-phosphate synthase L chain, ATP-binding:carbamoyl-phosphate synthetase l	No expression	++	+
*Brucella melitensis*	YP_419065	Full length	Blue (type 1) copper domain:blue (type 1) copper protein:amicyanin:plastocyanin	No expression	+	−
*Brucella melitensis*	YP_419020	Full length	Glutamate decarboxylase α	Insoluble	+	−
*Brucella melitensis*	YP_415432	Full length	Thioredoxin:thioredoxin type domain:thioredoxin domain 2	Soluble	++	ND
*Burkholderia pseudomallei*	YP_333769	Full length	Thioredoxin 1	Soluble	++	++
*Burkholderia pseudomallei*	YP_334416.1	Full length	Chorismate mutase	No expression	++	++
*Burkholderia pseudomallei*	ZP_04891863.1	267–404	Hemagglutinin-family protein	Insoluble	++	++
*Burkholderia pseudomallei*	YP_331616.1	Full length	Branched-chain amino-acid ABC transporter, ATP-binding protein	Insoluble	++	+++
*Burkholderia pseudomallei*	YP_334791.1	Full length	30S ribosomal protein S9	No expression	−	−
*Burkholderia pseudomallei*	YP_334870	Full length	3-Dehydroquinate dehydratase	Insoluble	+	+++
*Burkholderia pseudomallei*	YP_332852	Full length	NADH dehydrogenase subunit I	Insoluble	+	+++
*Burkholderia pseudomallei*	YP_334097	Full length	Phosphopyruvate hydratase	Insoluble	+	ND
*Burkholderia pseudomallei*	ABA48070.1	Bp 90–510	Sensor histidine kinase	Insoluble	++	++
*Burkholderia pseudomallei*	YP_333873	Full length	ATP-dependent protease ATP-binding subunit	No expression	+	−
*Burkholderia pseudomallei*	YP_334398.2	Full length	Adenosine deaminase	Soluble	+++	ND
*Burkholderia pseudomallei*	YP_334535.1	Full length	dTDP-glucose 4,6-dehydratase	Soluble	++	ND
*Burkholderia pseudomallei*	YP_333748	Full length	Histidyl-tRNA synthetase	Soluble	−	−
*Ehrlichia ruminantium*	YP_196632.1	Bp 96–891	FAD-dependent thymidylate synthase	ND	+	++
*Ehrlichia ruminantium*	YP_196632.1	Bp 96–762	FAD-dependent thymidylate synthase	ND	+	++
*Ehrlichia ruminantium*	YP_196632.1	Bp 96–654	FAD-dependent thymidylate synthase	ND	++	+
*Leishmania infantum*	XP_001464664.1	Full length	Elongation factor 1α	Insoluble	+	−
*Mycobacterium tuberculosis*	NP_216165.1	Full length	Phenylalanyl-tRNA synthetase subunit α	Insoluble	+++	+
*Mycobacterium tuberculosis*	NP_216786.1	Bp 57–525	Lipoprotein lppN	ND	++	+++
*Mycobacterium tuberculosis*	NP_217463.1	Full length	Probable polyketide synthase PKS15	No expression	++	++
*Rickettsia conorii*	NP_360910.1	Bp 3219–4419	190 kDa cell-surface antigen	Insoluble	+++	++
*Rickettsia prowazekii*	AAF34121.1	Bp 1–1095	OmpB	Insoluble	++	+
*Rickettsia prowazekii*	NP_221145	Full length	NADH dehydrogenase subunit I	Insoluble	ND	ND
*Rickettsia rickettsii*	P15921.1	3900–4953	Outer membrane protein A	Insoluble	++	+++
*Rickettsia rickettsii*	YP_001650445.1	Full length	Outer membrane protein B	ND	++	+
*Rickettsia rickettsii*	AAQ82709.1	3900–4953	Outer membrane protein A	ND	+	+
*Trypanosoma brucei*	XP_822456.1	Full length	Hexokinase	Insoluble	+	−

**Table 2:** Summary of protein-expression level and solubility from the cell-free expression system using small-scale expression, the Protemist DT II and the Protemist XE robotic platforms Conditions included WEPRO1240H and 7240H cell-free extracts in the presence and absence of NVoy. 1240H, WEPRO1240H extract; 7240H, WEPRO7240H extract; (−), without NVoy; (+), with 1 mg ml^−1^ NVoy.

	Small-scale screening		Protemist DT II				Protemist XE			
	1240H, (−)	1240H, (+)	1240H, (−)	1240H, (+)	7240H, (−)	7240H, (+)	1240H, (−)	1240H, (+)	7240H, (−)	7240H, (+)
No. of targets	44	—	29	5	6	5	4	1	4	2
Any expression (%)	87	—	97	80	67	80	100	100	100	100
Soluble expression (%)	56	—	59	80	17	80	75	100	50	100

**Table 3:** Summary of large-scale expression data in the presence or absence of NVoy Numbers indicate the numbers of milligrams of soluble protein purified in a 1 ml reaction cup on the Protemist DT II from each run. (+) and (−) indicate the presence or absence of NVoy at a concentration of 1 mg ml^−1^.

					NVoy (+):NVoy (−) ratio		7240H:1240H ratio	
Protein	1240H, NVoy (−)	1240H, NVoy (+)	7240H, NVoy (−)	7240H, NVoy (+)	1240H	7240H	NVoy (−)	NVoy (+)
*Rickettsia conorii* NP_360910.1, amino acids 1073–1473	0.37	0.91	0.96	1.72	3	2	3	2
*Ehrlichia ruminantium* YP_196632.1, amino acids 32–297	0.07	0.15	0.07	0.21	2	3	1	1
*Rickettsia prowazekii* AAF34121.1, amino acids 1–365	0.21	0.39	0.22	0.48	2	2	1	1
*Burkholderia pseudomallei* ZP_04891863.1, amino acids 267–404	0.11	0.26	0.13	0.60	2	5	1	2
*Leishmania infantum* XP_001464664.1	0.24	0.47	0.26	ND	2	ND	1	ND
*Trypanosoma brucei* XP_822456.1	0.27	0.28	0.28	0.61	1	2	1	2
